# Alcohol Consumption and Risk of Atrial Fibrillation: A Dose-Response Meta-Analysis of Prospective Studies

**DOI:** 10.3389/fcvm.2022.802163

**Published:** 2022-02-24

**Authors:** Heng-Zhi Zhang, Bo Shao, Qi-Yu Wang, Yi-Han Wang, Ze-Zhong Cao, Lu-Lu Chen, Jin-Yu Sun, Mu-Feng Gu

**Affiliations:** ^1^Department of Cardiology, The First Affiliated Hospital of Nanjing Medical University, Nanjing, China; ^2^School of Mathematical Science, University of Nottingham, Nottingham, United Kingdom; ^3^Department of Anatomy, Histology, and Embryology, Nanjing Medical University, Nanjing, China

**Keywords:** alcohol, alcohol consumption, atrial fibrillation, meta-analysis, low alcohol intake

## Abstract

**Background:**

This study aimed to investigate the dose-response association between alcohol consumption and atrial fibrillation (AF) risk.

**Methods:**

PubMed, Embase, Cochrane Library, and Web of Science were systematically searched using keywords related to alcohol and AF from the establishment of databases up to 1 March 2021. Prospective studies examining the impact of alcohol on the risk of AF with hazard ratios (HRs) were included. Restricted cubic spline regression was performed to quantify the dose-response relationship between alcohol consumption and AF risk.

**Results:**

Thirteen eligible studies were included in the meta-analysis, with a total of 645,826 participants and 23,079 cases of AF. When compared with non-/seldom-drinkers, the pooled adjusted HRs of AF were 1.30 (95% confidence interval [CI]: 1.20–1.41) and 1.00 (95% CI: 0.96–1.05) for high and low alcohol consumption, respectively. Moderate alcohol intake significantly increased the risk of AF in males (HR, 1.21; 95% CI: 1.10–1.33) but not in females (HR, 1.02; 95% CI: 0.91–1.14). The cubic spline regression analysis illustrated that the risk of AF significantly increased with daily alcohol intake in a Non-linear manner (R^2^ = 0.64, *P* = 5.785 × 10^−12^).

**Conclusion:**

This study revealed a Non-linearly positive association between alcohol intake and the risk of AF. Low alcohol intake was not associated with the development of AF, whereas moderate alcohol intake significantly increased the risk of AF in males but not in females. Our meta-analysis highlighted that alcohol consumption should be restricted to a low level to reduce the risk of AF.

## Introduction

Atrial fibrillation (AF) is the most common sustained cardiac arrhythmia, which results in a 4- to 5- fold increased risk for stroke, tripling of the risk for heart failure, and 40–90% higher risk for all-cause mortality (1). Previous studies have revealed multiple risk factors for AF, including older age, male sex, concomitant cardiovascular conditions, diabetes, and sleep apnea (2, 3). Excessive alcohol consumption has been demonstrated as a significant risk factor for multiple adverse health outcomes, including cardiomyopathy, hypertension, stroke (4), and cancer (5–7). Interestingly, several studies suggested potential protective effects of low-to-moderate alcohol consumption on the cardiovascular system (8, 9).

Although high-dose alcohol consumption has long been revealed as a risk factor for AF (10), whether low-to-moderate alcohol intake could elevate AF risk remains controversial (11–14). Several previous studies indicated a Non-significant relationship between low-to-moderate alcohol drinking and AF development (12–14), but a recent community-based study found that a modest drinking level (12 g per day) was associated with a higher risk of AF (11).

The findings of previous meta-analyses on the same topic are also inconsistent (15–18). One study (15) failed to conclude the impact of low alcohol intake on AF risk, and others (16–18) varied in the result concerning light drinking. No previous studies calculated dose-response meta-analysis using hazard ratios (HRs), which take time factors into account and offer more comprehensive results for time-to-event data. Since the last printed meta-analysis on this field, many new original studies have been published with more participants and advanced methods (11, 12, 14). Therefore, this dose-response meta-analysis aims to further investigate the dose-response relation between alcohol consumption and risk of AF using updated data and methods, with a focus on the low-to-moderate level of intake.

## Methods

### Search Strategy

A comprehensive literature search was conducted using online databases (including PubMed, Embase, Cochrane Library, and Web of Science) from the establishment of databases to 1 May 2021. We applied the search strategy as follows: (Atrial fibrillation OR AF OR flutter) AND (alcohol^*^ OR drink^*^ OR ethanol). Reference lists of selected articles were manually searched to ensure that all relevant papers had been identified.

### Study Designs

Clinical studies were eligible if they met the following inclusion criteria: (1) prospective cohort studies; (2) included only participants who were free of AF or atrial flutter (AFL) at baseline; (3) reported alcohol consumption as an exposure; (4) reported AF or a combination of AF/AFL as an outcome; (5) reported HRs with 95% confidence intervals (CIs) (or information allowing for their calculation) for at least three categories of alcohol consumption besides the reference group; (6) published in English. Additionally, we only included the publication with the most available data for multiple publications from the same trial or cohort.

### Data Extraction and Quality Assessment

Two researchers (B Shao and HZ Zhang) independently extracted data from each eligible study. Any disagreement was resolved by discussion or consulting a third researcher (JY Sun) until the consensus was reached. Data extracted from relevant publications included: first author, publication year, country, study design, study population, the proportion of females, age (mean and range), whether participants with heart disease excluded, the number of participants and cases, methods for assessment of alcohol consumption, methods of AF diagnosis, categories of alcohol consumption, follow-up duration, adjustments for confounding factors in generating the effect estimates.

The quality of studies involved was assessed by two researchers (B Shao and HZ Zhang) independently using the Newcastle-Ottawa Quality Assessment Scale (NOS). In this scale, the quality of studies was graded as poor (score <5), fair (score 5–7), and good (score > 8). Any disagreement was resolved by discussion or consulting a third researcher (JY Sun) until the consensus was reached.

### Statistical Analysis

The categorical meta-analysis was conducted using STATA software version 16. The hazard ratios of developing AF extracted from the included studies were pooled with 95% CIs. The heterogeneity between studies was assessed based on Q statistics and I-squared. A common-effect inverse-variance model was applied for calculating pooled estimates if the heterogeneity was not statistically significant (I^2^ ≤ 50%, *P* ≥ 0.05). Otherwise, a random-effects inverse-variance model with DerSimonian-Laird estimate of tau^2^ was applied (19). We graphed funnel plots and conducted Duval and Tweedie's trim and fill procedure to estimate the influence of potential publication bias (20). In addition, Egger's regression asymmetry test was used for further quantitative evaluation of the possibility of publication bias (21). Publication bias existed if *P* < 0.10 (22). For sensitivity analysis, a one-study removal analysis (i.e., omit one study sequentially in the calculation of the pooled estimate to test the stability and reliability of the result) was conducted to assess the influence of each study on the pooled estimate.

Dose-response meta-analysis was conducted using R software with pracma, car, and ggplot2 packages. To minimize the overlapping of alcohol intake groups of different studies, we limited the dose-response analysis to studies that met two criteria: (1) the alcohol intake of <1.72 g/day (12 g/week) was set as the reference category, and (2) at least three alcohol consumption categories besides the reference group were reported in the study. Initially, a linear regression model based on the weighted least-square method was used to explore the dose-response relationship between daily alcohol consumption and the risk of AF. To further explore the shape of the relationship, we applied restricted cubic spline regression with 4 knots at the minimum value, 25^th^, 75^th^ centiles, and maximum value of the distribution of alcohol consumption (23). A 2-tailed value of *P* < 0.05 was considered statistically significant.

## Results

### Study Selection and Characteristics

**Figure 1:**
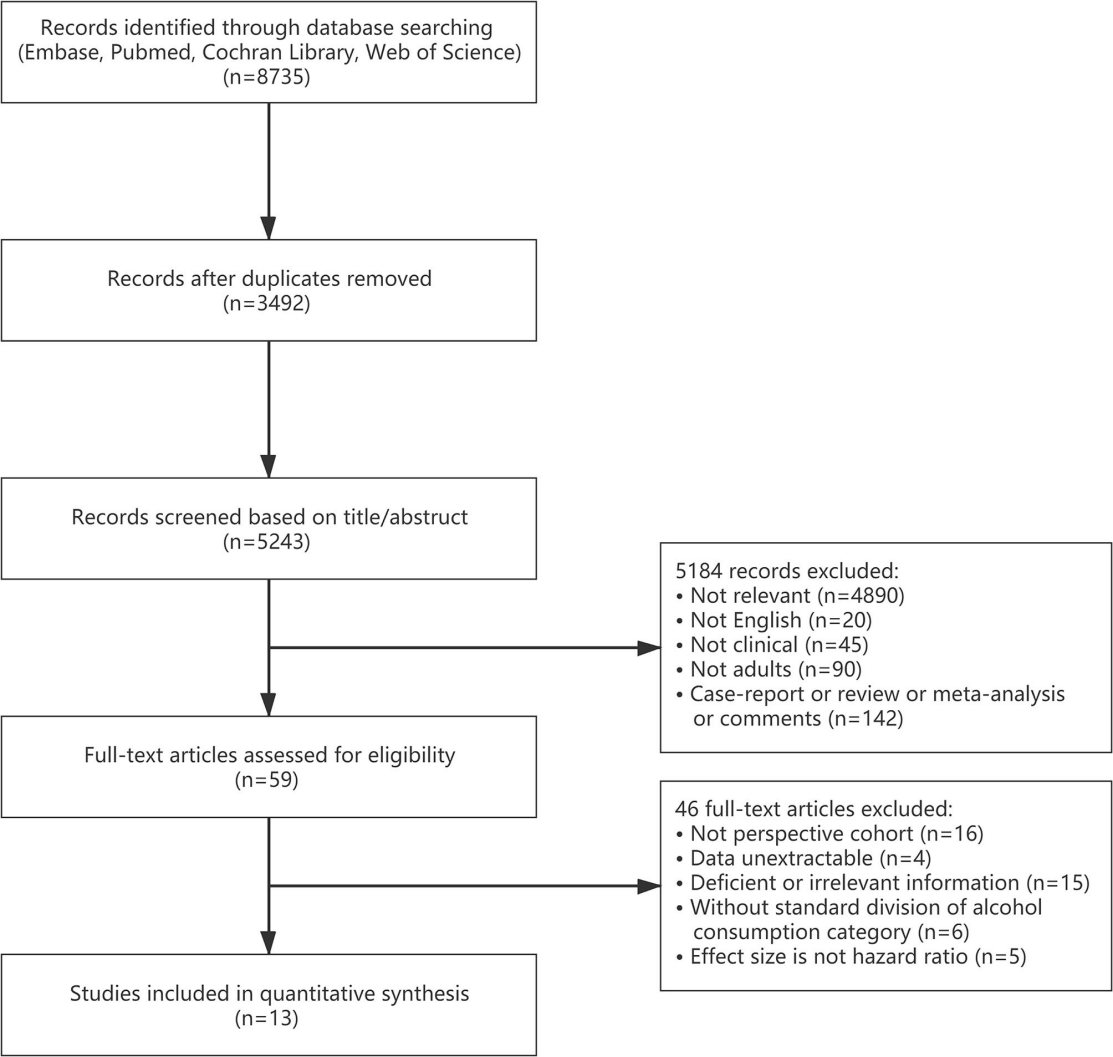
The flow chart of the study selection process.

The flowchart of the study selection process is shown in Figure 1. A total of 8,735 articles were identified in the initial search from Embase, PubMed, Cochran Library, and Web of Science. We removed 3,492 duplicates and excluded a further 5,184 studies after screening titles and abstracts, leaving 59 articles retrieved for full-text screening. Then, 46 articles were excluded for reasons outlined in Figure 1. Finally, a total of 13 studies (11–14, 24–32) comprising 645,826 participants were included.

Among the 13 included studies, 3 (23.1%) studies were conducted in North America, 8 (61.5%) in Europe, 1 (7.7%) in Asia, and 1 (7.7%) were in 40 countries (from Europe, America, Australia, and Asia). All the studies were prospective cohort studies. Four studies (12, 24, 26, 28) excluded participants with heart disease, and the remaining nine studies (11, 13, 14, 25, 27, 29–32) made adjustments for heart disease. Additionally, five studies reported gender subgroup analyses. Characteristics of included studies are summarized in Table 1. The scores of included studies assessed by NOS were ranged from 6 to 9 (Table 2), which means all included articles were regarded as moderate or high quality.

**Table 1:** Characteristics of the studies included in the meta-analysis.

**Study**	**Country**	**Study population**	**% Female**	**Age (years), range (mean)**	**Patients with heart disease excluded**	**Number of participants**	**Follow-up duration (years)**
Frost et al. (28)	Denmark	Population-based, the Danish Diet, Cancer and Health Study	53	50–64 (51)	Yes	47,949	5.7 (median)
Mukamal et al. (24)	Denmark	Population-based, the Copenhagen City Heart Study	54	Men: 26–75 (51) Women: 26–73 (52)	Yes	16,415	Men:16.6 Women:18.3
Mukamal et al. (25)	US	Population-based, the Cardiovascular Health Study	56	≥65 (73.1)	No	5,609	9.1
Conen et al. (26)	US	Population-based, the Women's Health Study	100	≥45 (53)	Yes	34,715	12.4 (median)
Shen et al. (13)	US	Population-based, the Framingham Heart Study	56.1	≥45 (62)	No	4,526	4
Liang et al. (29)	40 countries worldwide	Participants of the ONTARGET and TRANSCEND trials	29.8	≥55 (66.4)	No	30,433	4.7 (median)
Sano et al. (30)	Japan	Population-based, the Circulatory Risk in Communities Study (CIRCS)	64.2	30–80 (57.1)	No	7,206	6.4 (median)
Tolstrup et al. (31)	Denmark	Population-based, the Copenhagen City Heart Study and the Copenhagen General Population Study	54.9	48–67 (58)	No	88,782	6.1 (mean)
Di Castelnuovo et al. (12)	Italy	Population-based, the Moli-sani study	51.9	≥35 (55)	Yes	22,628	8.2 (median)
Gémes et al. (27)	Norway	Population-based, the Norwegian HUNT Study	55	≥20 (52.3)	No	47,002	8
Bazal et al. (14)	Spain	Participants of the PREDIMED trial	56.8	55–80 (67)	No	6,077	4.4
Ariansen et al. (32)	Norway	Norwegian population-based health examination surveys	51	≥40 (43.5)	No	234,392	9
Csengeri et al. (11)	Sweden	Five community-based cohorts	51.7	37.8–58.6 (47.8)	No	100,092	13.9 (median)
**Study**	**Method for assessment of exposure**	**Method of AF diagnosis**	**Categories of alcohol consumption**	**Adjustments**
Frost et al. (28)	Structured questionnaires	Registries	Men:4.1 ± 2.6, 12.1 ± 2.1, 20.0 ± 3.0, 36.1± 4.9, 68.7 ± 22.8 g/day Women: 1.1± 0.7, 4.6 ± 1.5, 9.4 ±1.7, 15.6 ±2.6, 38.8 ±14.8 g/day	Age, height, BMI, smoking, systolic BP, treatment for hypertension, total serum cholesterol, education
Mukamal et al. (24)	Standardized interviews	ECG screening, medical records, registries	Men: <1, 1–6, 7–13, 14–20, 21–27, 28–34, ≥35 drinks/week Women: <1, 1–6, 7–13, 14–20, ≥21 drinks/week	Age, smoking, education, cohabitation, family history of CVD, diabetes, income, physical activity, BMI, FEV_1_, height, use of BP medication, systolic BP, incident diagnoses of CHD or CHF
Mukamal et al. (25)	Structured questionnaires	ECG screening, medical records, registries	None, former, 1–6, 7–13, ≥14 drinks/week	Age, sex, BMI, smoking, systolic BP, race, income, height, waist, circumference, depressive symptom score, drug usage, physical activity, psychoactive medication, diabetes, hypertension, CHD, congestive heart failure and total cholesterol
Conen et al. (26)	Structured questionnaires	Self-reported confirmed by medical record	None, <1, 1–2, ≥2 drinks/day	Age, race, BMI, smoking status, systolic BP, history of hypertension and hypercholesterolemia, diabetes, hypercholesterolemia, exercise, education
Shen et al. (13)	Structured questionnaires	ECG screening	None, 1–3, 3–13, 13–161 g/day	Age, sex, BMI, systolic blood pressure, hypertension treatment, electrocardiogram, PR interval, significant heart murmur, and heart failure
Liang et al. (29)	Questionnaires	ECG screening	Low <1 drink per week, moderate 1–14 drinks for women and 1–21 drinks for men, high >2 drinks per day for women and >3 drinks per day for men, binge drinkers >5 drinks per day	Age, sex, BMI, smoking status, region, medical history of CAD, stroke of TIA, hypertension, diabetes, chronic renal disease and sleep apnea, education, exercise, use of statin and treatment allocation (ramipril, telmisartan or both vs. placebo)
Sano et al. (30)	Interviews	ECG screening, hospital reports	Never, past, <23, 23–45, 46–69, >69 g/day	Age, sex, cigarette smoking status, BMI, hypertension, hyperglycemia, hyperlipidemia, major ST-T abnormality, previous myocardial infarction, and heart failure
Tolstrup et al. (31)	Self-administered questions	ECG screening, medical records, registries	Man: <1, 1–6, 7–13, 14–20, 21–27, 28–34, ≥35 drinks/week Women: <1, 1–6, 7–13, 14–20, 21–27, ≥28 drinks/week	Age, sex, BMI, smoking status, height, diabetes, education, living alone, FEV/FVC, hypertension, family history of CVD, angina, CHD, heart medication, use of cholesterol-lowering drugs
Di Castelnuovo et al. (12)	Structured questionnaires	Medical records	Former, never, occasional, 1–12, 12.1–24, 24.1–48, >48 g/day	Age, sex, smoking, education, income, physical activity, body mass index, total cholesterol, total calorie intake, history of cardiovascular disease, hypertension, and diabetes
Gémes et al. (27)	Self-administered questions	Registries	Abstainers, rare drinkers, >0 and ≤ 3, >3 and ≤ 7, >7 drinks/week	Age, sex, BMI, smoking status, physical activity, living in a relationship, previous CVD, any chronic disease, cholesterol, HDL-C, BP, anxiety score, depression score
Bazal et al. (14)	Structured questionnaires	ECG screening, medical records	Non-drinkers, low-moderate <30 g/day in men and <15 g/day in women, Mediterranean pattern, heavy drinkers ≥30 g/day in men and ≥15 g/day in women	Age, sex, intervention group, smoking, body mass index, height, physical activity, sleep apnea, depression, diabetes, diastolic and systolic blood pressure, hypertension, Non-atherosclerotic coronary disease, and heart failure
Ariansen et al. (32)	Questionnaires	Registries	<2, 2–12, 12–24, ≥24 g/day	Age, sex, education, marital status, smoking, physical activity, body mass index, resting heart rate, total cholesterol concentration, triglyceride concentration, diabetes, family history of coronary heart disease, and history of cardiovascular disease
Csengeri et al. (11)	Questionnaires	Questionnaires, registries	<1, 1–12, 12.1–24, 24.1–48, >48 g/day	Age, sex, BMI, hypertension, systolic blood pressure, diabetes, current daily smoker, anti-hypertensive medication, history of heart failure, myocardial infarction or stroke, employment status, education

*BMI, body mass index; BP, blood pressure; CVD, cardiovascular disease; CHD, coronary heart disease; FEV_1_, forced expiratory volume in one second; FVC, forced vital capacity; CHF, congestive heart failure; TIA, transient ischemic attack*.

**Table 2:** Quality assessment using the Newcastle-Ottawa Scale for cohort studies.

**Study**	**Selection**				**Comparability**	**Outcome**			**Scores**
	**Representativeness of the exposed cohort**	**Selection of the Non-exposed cohort**	**Ascertainment of exposure**	**Demonstration that outcome of interest was not present at start of study**	Comparability of cohorts on the basis of the design or analysis^*^	**Assessment of outcome**	**Was follow-up long enough for outcomes to occur**	**Adequacy of follow up of cohorts**	
Frost et al. (28)	⋆	⋆	⋆	⋆	⋆⋆	⋆	⋆	⋆	9
Mukamal et al. (24)	⋆	⋆	⋆	⋆	⋆⋆	⋆	⋆	–	8
Mukamal et al. (25)	–	⋆	⋆	⋆	⋆⋆	⋆	⋆	–	7
Conen et al. (26)	⋆	⋆	–	⋆	⋆⋆	–	⋆	⋆	7
Shen et al. (13)	⋆	⋆	–	⋆	⋆⋆	⋆	⋆	–	7
Liang et al. (29)	–	⋆	⋆	⋆	⋆⋆	⋆	⋆	⋆	8
Sano et al. (30)	⋆	⋆	⋆	⋆	⋆⋆	⋆	⋆	⋆	9
Tolstrup et al. (31)	⋆	⋆	⋆	⋆	⋆⋆	⋆	⋆	–	8
Di Castelnuovo et al. (12)	⋆	⋆	⋆	⋆	⋆⋆	⋆	⋆	⋆	9
Gémes et al. (27)	⋆	⋆	–	⋆	⋆⋆	⋆	⋆	⋆	8
Bazal et al. (14)	⋆	⋆	⋆	⋆	⋆⋆	⋆	⋆	–	8
Ariansen et al. (32)	⋆	⋆	–	–	⋆⋆	⋆	⋆	–	6
Csengeri et al. (11)	⋆	⋆	–	⋆	⋆⋆	⋆	⋆	–	7

* *A maximum of 2 stars can be allotted in this category, one for age, the other for other controlled factors*.

### Assessment of Alcohol Consumption

Alcohol intake was assessed by questionnaire in 9 studies, interview in 2 studies, and self-administered question in 2 studies (Table 1). To standardize alcohol intake, we used a common scale (grams per day) for ethanol consumption. When a study used the number of drinks per day as a unit of alcohol intake, the unit was transformed into grams of ethanol according to the study-specific methods for estimating the amount of ethanol per drink. If the amount of ethanol per drink was not specified, the unit was considered equivalent to 12 g ethanol (33). Importantly, the reference groups were slightly different: 6 studies set individuals without alcohol intake as a reference, whereas seldom-drinkers (<2 g per day) were used in the remaining seven ones. Moreover, we divided alcohol consumption into three groups based on the following criteria: (1) if the study has divided alcohol consumption into low, moderate, and high groups, use those groups; (2) if there are more than three study categories apart from the reference group, the highest category is assigned to the high alcohol intake group, the lowest to the low alcohol intake group, and the closest to 12 to 24 g/day is assigned to the moderate alcohol intake group.

### Categorical Meta-Analysis

#### High Alcohol Intake

**Figure 2:**
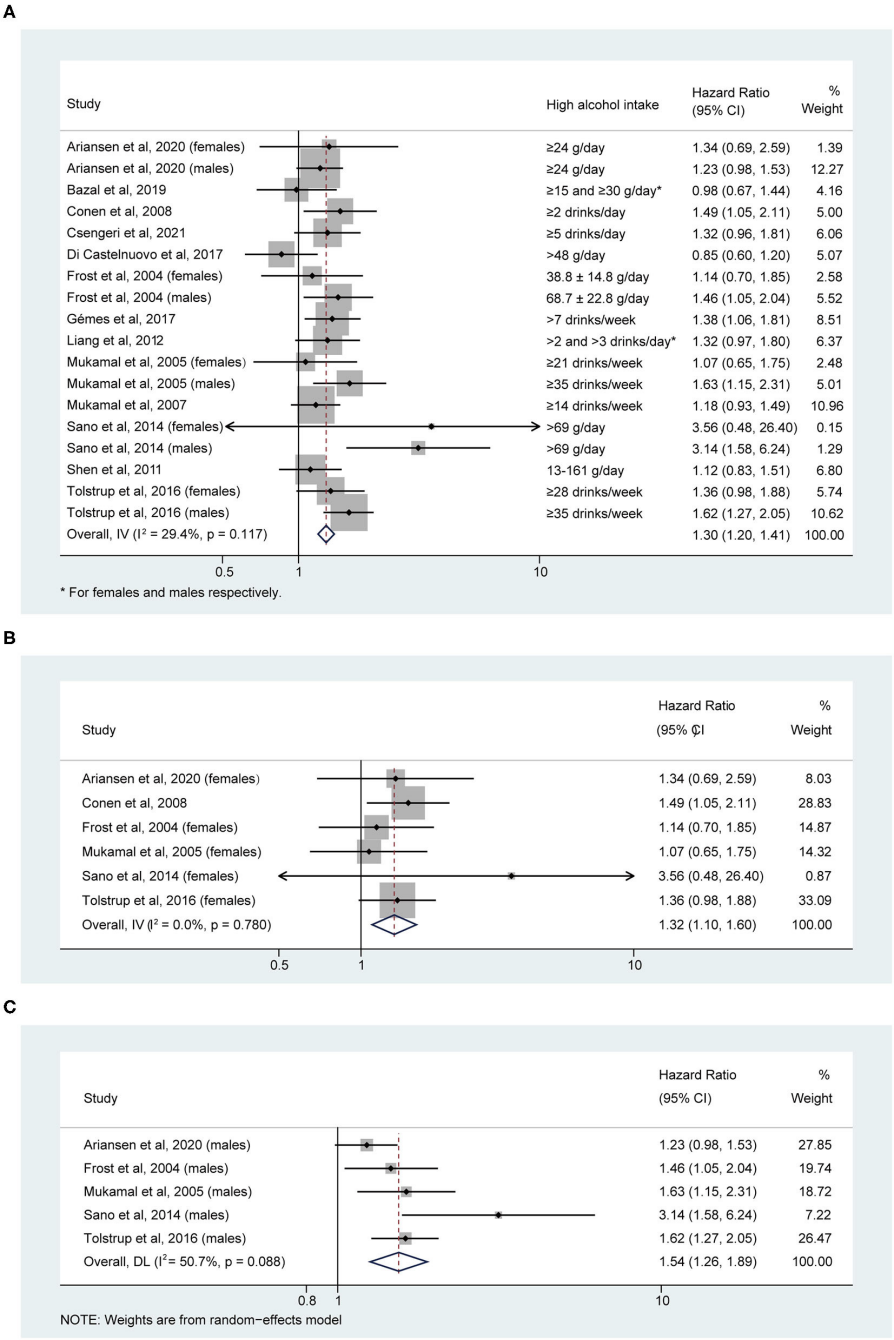
Hazard ratios of risk of atrial fibrillation for high alcohol intake in **(A)** overall, **(B)** female, and **(C)** male individuals.

Compared to the reference group (Table 1), high alcohol consumption significantly increased the risk of AF development, with a pooled HR of 1.30 (95% CI: 1.20–1.41; Figure 2A). There was no significant heterogeneity between studies (I^2^ = 29.4%, *P* = 0.117). The relation between high alcohol intake and higher risk of AF was significant for both females (HR, 1.32; 95% CI: 1.10–1.60; Figure 2B) and males (HR, 1.54; 95% CI: 1.26–1.89; Figure 2C). There was evidence of heterogeneity between studies for males (I^2^ = 50.7%, *P* = 0.088) but not females (I^2^ = 0.0%, *P* = 0.780).

Egger's test indicated no significant publication bias (*t* = 0.83, *P* = 0.417), while the funnel plot asymmetry indicated publication bias (Supplementary Figure 1A). Then, we used the trim and fill method (20) to adjust the publication bias. After incorporating one unpublished result to produce a hypothetically symmetrical funnel plot, no alteration was observed in the pooled estimate (HR, 1.30; 95% CI: 1.20–1.41). Next, we limited the analysis to 7 studies that regarded only Non-drinkers as the reference group for sensitivity analysis. The pooled HR remained 1.24 (95% CI: 1.02–1.52), while there was a higher heterogeneity between studies (I^2^ = 57.0%, *P* = 0.023). Then, the one-study removal analysis was conducted to estimate the stability of the meta-analysis further. We omitted one study sequentially, and the pooled estimate still indicated a significant relationship between high alcohol intake and a higher risk of AF (Supplementary Figure 2A).

#### Moderate Alcohol Intake

**Figure 3:**
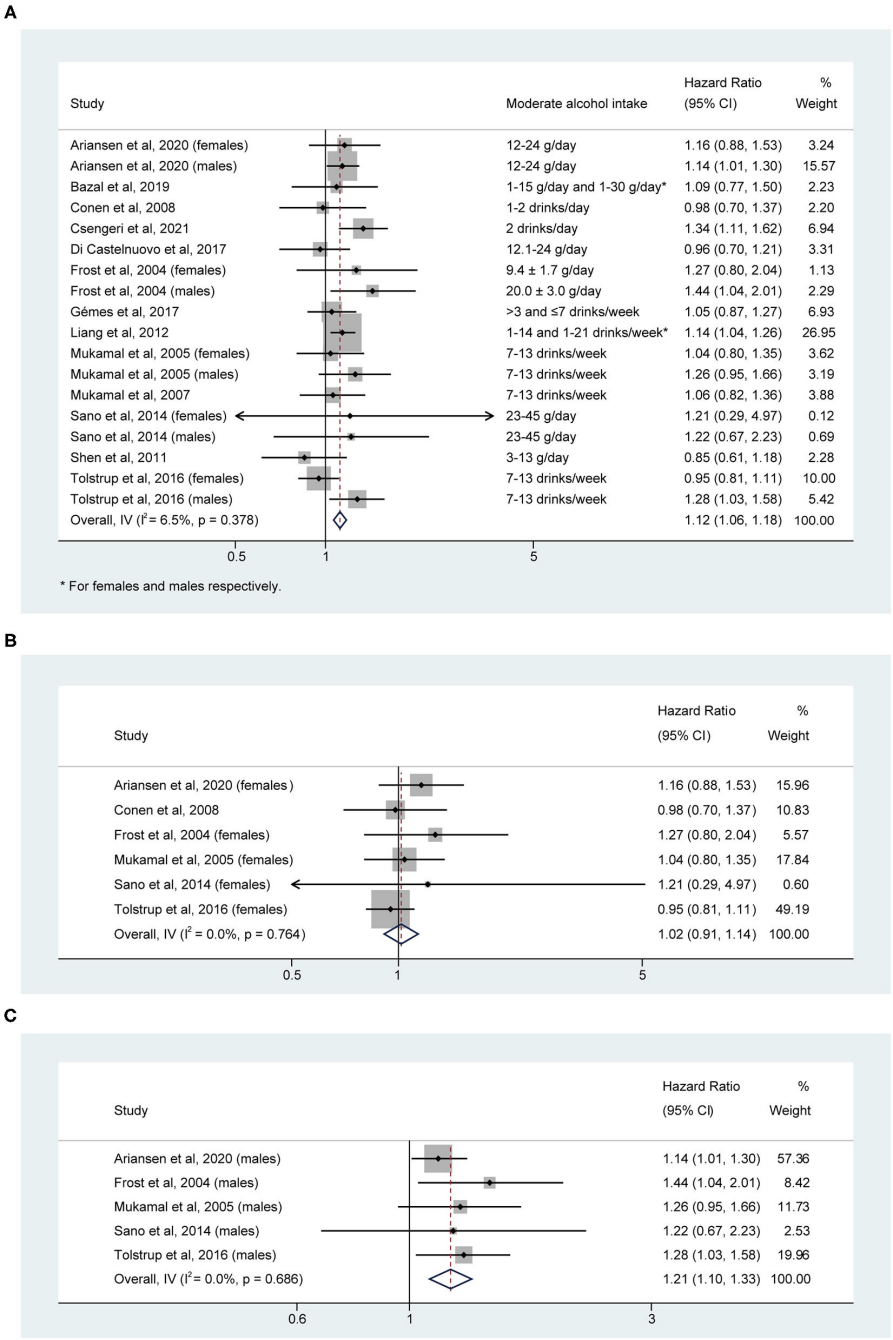
Hazard ratios of risk of atrial fibrillation for moderate alcohol intake in **(A)** overall, **(B)** female, and **(C)** male individuals.

Moderate alcohol intake (mostly defined as 12–24 g/day) was associated with a small but still significant increase in the risk of AF (HR, 1.12; 95% CI: 1.06–1.18; Figure 3A). However, there was no significant relation between moderate alcohol intake and higher risk of AF for females (HR, 1.02; 95% CI: 0.91–1.14; I^2^ = 0.0%, *P* = 0.764; Figure 3B). The pooled HR for males was 1.21 (95% CI: 1.10–1.33) with no evidence of heterogeneity between studies (I^2^ = 0.0%, *P* = 0.686; Figure 3C).

Evidence of publication bias was visually suggested by the asymmetry of the funnel plot (Supplementary Figure 1B). The trim and fill method was then performed to adjust the publication bias. The pooled estimate was not significantly altered (HR, 1.11; 95% CI: 1.06–1.17) after incorporating one negative unpublished result to produce a hypothetically symmetrical funnel plot. Egger's test indicated no evidence of publication bias (*t* = −0.03, *P* = 0.974). In sensitivity analysis, exclusion of six studies to limit the criteria for moderate alcohol intake to 12–24 g/day did not alter the result (HR, 1.12; 95% CI: 1.05–1.19) with no evidence of statistical heterogeneity (I^2^ = 26.7%, *P* = 0.198). One-study removal analysis suggested that no single study significantly altered the pooled results, indicating the stability of the results (Supplementary Figure 2B).

#### Low Alcohol Intake

**Figure 4:**
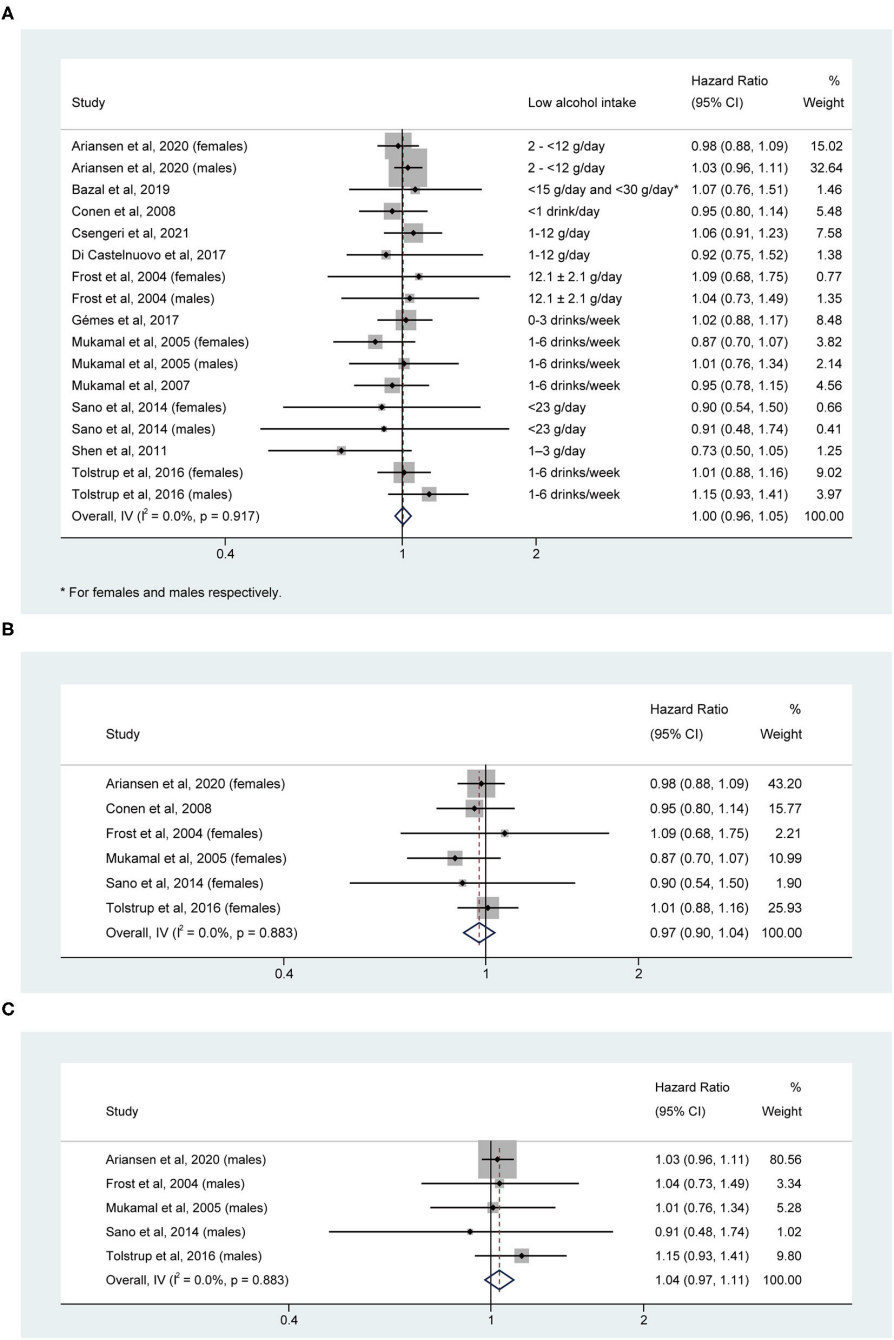
Hazard ratios of the risk of atrial fibrillation for low alcohol intake in **(A)** overall, **(B)** female, and **(C)** male individuals.

The association between low alcohol consumption and risk of AF was not significant (HR, 1.00; 95% CI: 0.96–1.05; I^2^ = 0.0%, *P* = 0.917; Figure 4A). The Non-significant result remained in the sensitivity analysis for each sex, with HRs of 0.97 (95% CI: 0.90–1.04) for females and 1.04 (95% CI: 0.97–1.11) for males. No significant heterogeneity between studies was observed (I^2^ = 0.0%, *P* = 0.883 for both sexes; Figures 4B,C).

Both funnel plot (Supplementary Figure 1C) and Egger's test (*t* = −1.31, *P* = 0.211) indicated little evidence of publication bias. We limited the analysis to seven studies that regarded only Non-drinkers or never-drinkers as the reference group for sensitivity analysis. The pooled estimate was still statistical Non-significant (HR, 0.96; 95% CI: 0.89–1.05) with little evidence of heterogeneity between studies (I^2^ = 0.0%, *P* = 0.853). One-study removal analysis also indicated that the pooled estimate was statistically stable and reliable (Supplementary Figure 2C).

### Dose-Response Meta-Analysis

**Figure 5:**
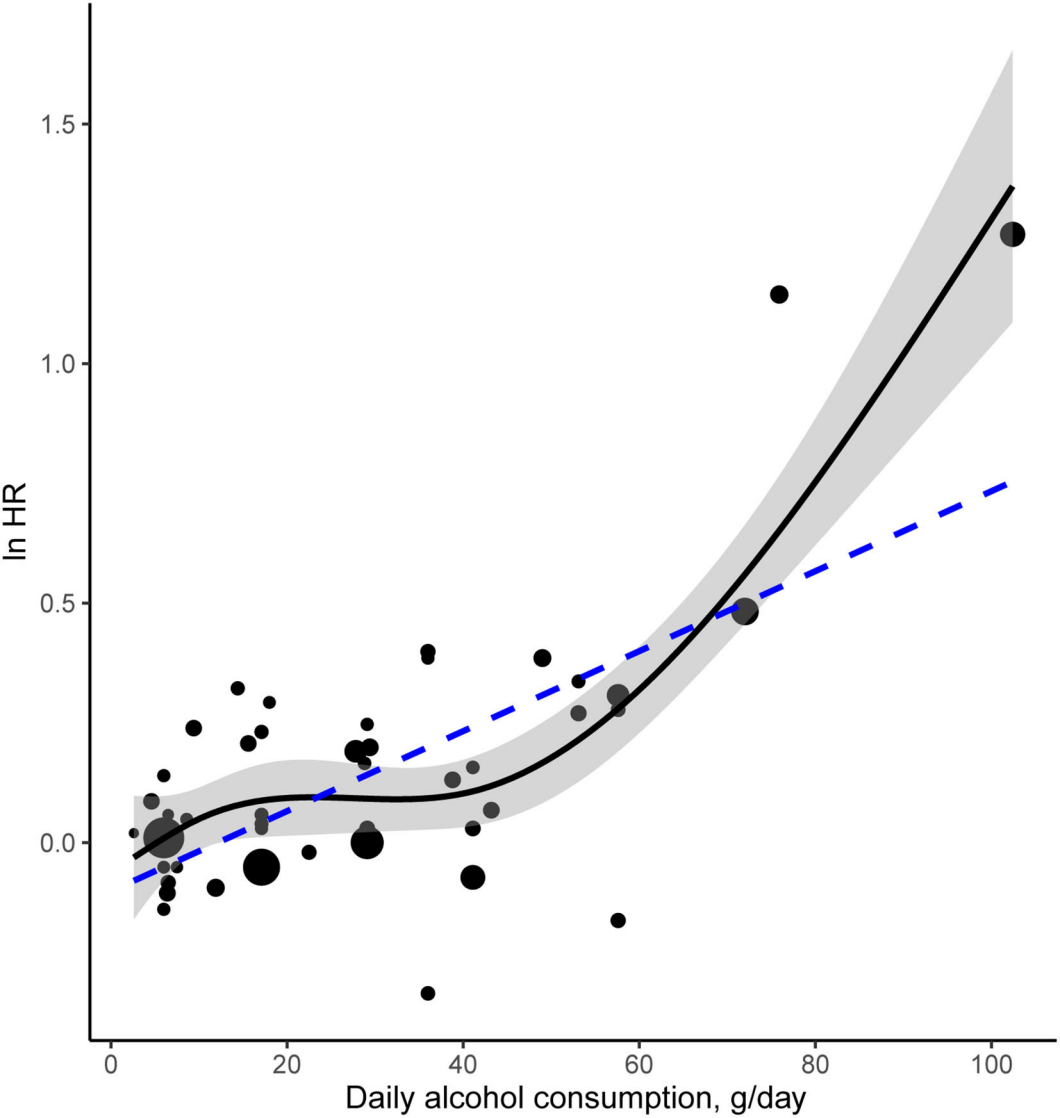
A dose-response meta-analysis of alcohol consumption and risk of AF. The solid curve and the shadow area indicate the ln(HR) with a corresponding 95% confidence interval based on a restricted cubic spline regression model with knots 2.6, 10.65, 41.1, 102.4 g/day of alcohol consumption. This model significantly improves the fit compared with the linear regression model illustrated by the dotted line. The area of each data point is proportional to its statistical weight.

Figure 5 illustrates the dose-response relationship between daily alcohol intake and the risk of AF. Seven studies involving 222,357 participants and 9,626 cases were eligible for analysis of the dose-response relationship. The linear curve indicated a significant relationship between daily alcohol intake and the risk of AF (R^2^ = 0.47, *P* < 0.001). The coefficient for the linear term was −0.101327. To fit the model precisely, restricted cubic spline regression was applied for the dose-response analysis of HR. The estimated HRs were 1.01 (95% CI: 0.92–1.10) at 6 g/day, 1.09 (95% CI: 1.01–1.29) at 18 g/day, 1.75 (95% CI: 1.58–1.94) at 72 g/day, and 3.94 (95% CI: 1.17–1.28) at 102.4 g/day. Compared to the linear regression model, cubic spline regression showed a smooth curve with a larger R square coefficient (R^2^ = 0.64, *P* = 5.785 × 10^−12^), indicating a decent fit for this model. The difference between the fit for the linear and cubic spline regression model is statistically significant (*P* < 0.01).

## Discussion

This study showed that high alcohol consumption was associated with a significantly increased risk of AF in both genders, which was consistent with previous studies (15–18). The association remains robust in the sensitivity analysis when we limited the analysis to 7 studies setting none- or never- drinkers as reference.

In addition, this study suggested a sex-related association between moderate alcohol intake and the risk of AF: moderate alcohol intake significantly increased the AF risk in males but not in females. Sex-related psycho-socio-cultural factors may account for the difference. Male drinking style is often public, less circumscribed, and more associated with aggressive behaviors (34–36). Conversely, women tend to drink at home or with a friend (37), prefer mild alcoholic beverages (e.g., wine and beer) (38, 39), have a more controlled drinking style, and become less frequently intoxicated (40). These characteristics might make women more likely to show better outcomes compared with men (41, 42). Additionally, sex-related alcohol metabolism and alcohol-sex hormone interactions might also result in differences. However, the studies on the underlying biological mechanisms yielded controversial results (43–45). A further investigation on the sex-related difference would be an interesting topic in the following research.

Moreover, our analysis revealed no association between low alcohol consumption and higher AF risk. The results remained stable when limiting the analysis to 7 studies that set only non- or never-drinkers as reference. Alcohol showed a dose-dependent effect on atrial electrical and structural changes, thus inducing the risk of AF. Voskoboinik, A. *et al*. (46) observed significant electrophysiological and structural changes in the atrial substrate in patients consuming 8–21 drinks/week but not in those consuming 1–7 drinks/week compared to Non-drinkers, which is in line with our findings. It is noteworthy that low alcohol consumption showed cardiovascular benefits against many cardiovascular diseases [including coronary heart disease, myocardial infarction, and stroke (47, 48)], possibly owing to the increased high-density lipoprotein cholesterol and anti-inflammatory effect (49, 50). However, the effect of low alcohol on AF remains vague (12, 51). Since AF shares many common risk factors with other cardiovascular diseases, those cardiovascular benefits might also counteract the electrical and structural changes caused by low alcohol intake.

In dose-response meta-analysis, the cubic spline regression showed a significantly better fit than the linear regression, indicating a Non-linear association of alcohol consumption with the risk of AF. The dose-response curve generally indicated a positive correlation between alcohol intake and the AF risk, characterized by an initial slight increase in risk at lower dosage followed by a steeper rise over ~40 g per day. A threshold theory may partially explain this phenomenon. We speculated that there was probably a threshold of alcohol intake, above which the toxic effect of ethanol would become more significant, following by the serious damage to the cardiac electrophysiological system. However, there lacks sufficient direct evidence to support this potential threshold.

The results of our study partially differ from previous meta-analyses on this topic. Two previous meta-analyses reported a small but significant increase in AF risk for low-level drinkers (16, 17), and the study by Kodama, S. *et al*. manifested a linear association between alcohol intake and the risk of AF (16). Study design and the included population may account for the difference. Our study restricted the inclusion criteria to only prospective cohort studies, while previous ones also incorporated retrospective or case-control design. The Non-cohort studies might lead to selection bias and thus overestimate the risk. Additionally, most studies included in the previous meta-analyses were from the United States or Northern Europe, where wine (with potential healthy nonalcoholic components) is less popular and the tendency of irregular drinking is high. Differently, we also included studies from Southern Europe and Asia, which provided a better global perspective on the role of alcohol. Still, further studies are required to assess whether a different effect of alcohol on AF exists across areas. Moreover, the number of participants included in our study (645,826) is larger than previous meta-analyses (ranged from 67,891 to 249,496), which further reduced the risk of random errors (15–18). Fourth, we only applied HRs, instead of relative risks or odd ratios as in the previous study (16), as effective sizes in our calculation of pooled estimates. With the time factor taken into account, this method could draw more comprehensive and convincing results with time-to-event data. For the difference in the dose-response analyses, another possible explanation is that the range of alcohol consumption analyzed in our study (up to 102.4 g/day) was wider compared with the previous one (up to about 86 g/day), making it possible for us to calculate a more precise meta-regression by the extended data.

Our study supported that low alcohol consumption (primarily 0–12 g/day) might be a potentially safe threshold for AF risk. Alcohol showed a dose-dependent effect on atrial electrical and structural changes, thus inducing the risk of AF. Voskoboinik, A. *et al*. (46) observed significant electrophysiological and structural changes in the atrial substrate in patients consuming 8–21 drinks/week but not in those consuming 1–7 drinks/week compared to Non-drinkers. Interestingly, low alcohol consumption showed cardiovascular benefits against many cardiovascular diseases [including coronary heart disease, myocardial infarction, and stroke (47, 48)] possibly owing to the increased high-density lipoprotein cholesterol and anti-inflammatory effect (49, 50). However, the effect of low alcohol on AF remains vague (12, 51). Since AF shares many common risk factors with other cardiovascular diseases, those cardiovascular benefits might also reduce the risk of AF.

One of the strengths of this meta-analysis is that we conducted cubic spline regression analyses to quantify the relationship between alcohol intake and AF risk beyond summarizing the association through categorical analyses. Moreover, our study restricted the inclusion criteria to prospective studies, thus minimizing the potential influence of recall and selection bias. To the best of our knowledge, this meta-analysis is the first to conduct both categorical and dose-response analysis for HRs of AF on alcohol consumption, which offers more comprehensive and convincing results with time-to-event data comparing to relative risks or odds ratios. In addition, we included a larger number of participants from several new studies comparing to previous meta-analyses on this field (15–18), making us able to conclude more convincing and updated results.

Despite the advantages, several limitations should be mentioned. First, the definition of low, moderate, and high drinking is heterogeneous across studies, and the reference groups are also not uniform. Still, we performed sensitivity analyses to alleviate this limitation. Second, the majority of the included studies did not state whether the method used to assess alcohol intake was validated. Third, adjustments for confounding factors are slightly different across the studies. Most of the studies adjusted for common confounders, including basic physical information and major cardiovascular risk factors, while a few of the studies adjusted for additional factors such as education, socio-economic status, and ethnicity, which may influence the pooled analyses in our study. Finally, the risk associated with each level of alcohol intake and the threshold at which this increases AF risk would be difficult to elicit without very detailed data collection from study participants, especially the threshold of low alcohol intake where the association becomes significant.

## Conclusions

High alcohol intake is associated with an increased risk of AF, but the association is not significant for low alcohol intake. The risk of AF rises in males but not in females for moderate alcohol intake. The dose-response relationship between daily alcohol intake and the risk of AF is Non-linearly positive. Our meta-analysis highlighted that alcohol consumption should be restricted to a low level to reduce the risk of AF.

## Data Availability Statement

The original contributions presented in the study are included in the article/Supplementary Material, further inquiries can be directed to the corresponding authors.

## Author Contributions

H-ZZ, L-LC, J-YS, and M-FG conceived and designed the study. H-ZZ, BS, Q-YW, and Y-HW analyzed the data. H-ZZ, BS, and Z-ZC wrote the paper. All authors provided critical revisions of the manuscript and approved the final manuscript.

## Funding

This study was supported in part by the Natural Science Foundation of Jiangsu Province (No. 21KJB320006), Postgraduate Research & Practice Innovation Program of Jiangsu Province (SJCX21_0626), and College Students Innovation and Entrepreneurship Training Program of Jiangsu Province (No. 202110312079Y).

## Conflict of Interest

The authors declare that the research was conducted in the absence of any commercial or financial relationships that could be construed as a potential conflict of interest.

## Publisher's Note

All claims expressed in this article are solely those of the authors and do not necessarily represent those of their affiliated organizations, or those of the publisher, the editors and the reviewers. Any product that may be evaluated in this article, or claim that may be made by its manufacturer, is not guaranteed or endorsed by the publisher.

## Supplementary Material

Supplementary Figure 1 Funnel plots for alcohol consumption and AF. **(A)** high alcohol intake, filled; **(B)** moderate alcohol intake, filled; **(C)** low alcohol intake.

Supplementary Figure 2 Sensitivity analysis using the one-study removal method. **(A)** high alcohol intake; **(B)** moderate alcohol intake; **(C)** low alcohol intake.
