# Supplementary figures and images for: Alcohol Consumption and Risk of Atrial Fibrillation: A Dose-Response Meta-Analysis of Prospective Studies
Source: Front Cardiovasc Med. 2022 Feb 24;9:802163. doi: 10.3389/fcvm.2022.802163 (PMC8907587; doi:10.3389/fcvm.2022.802163)

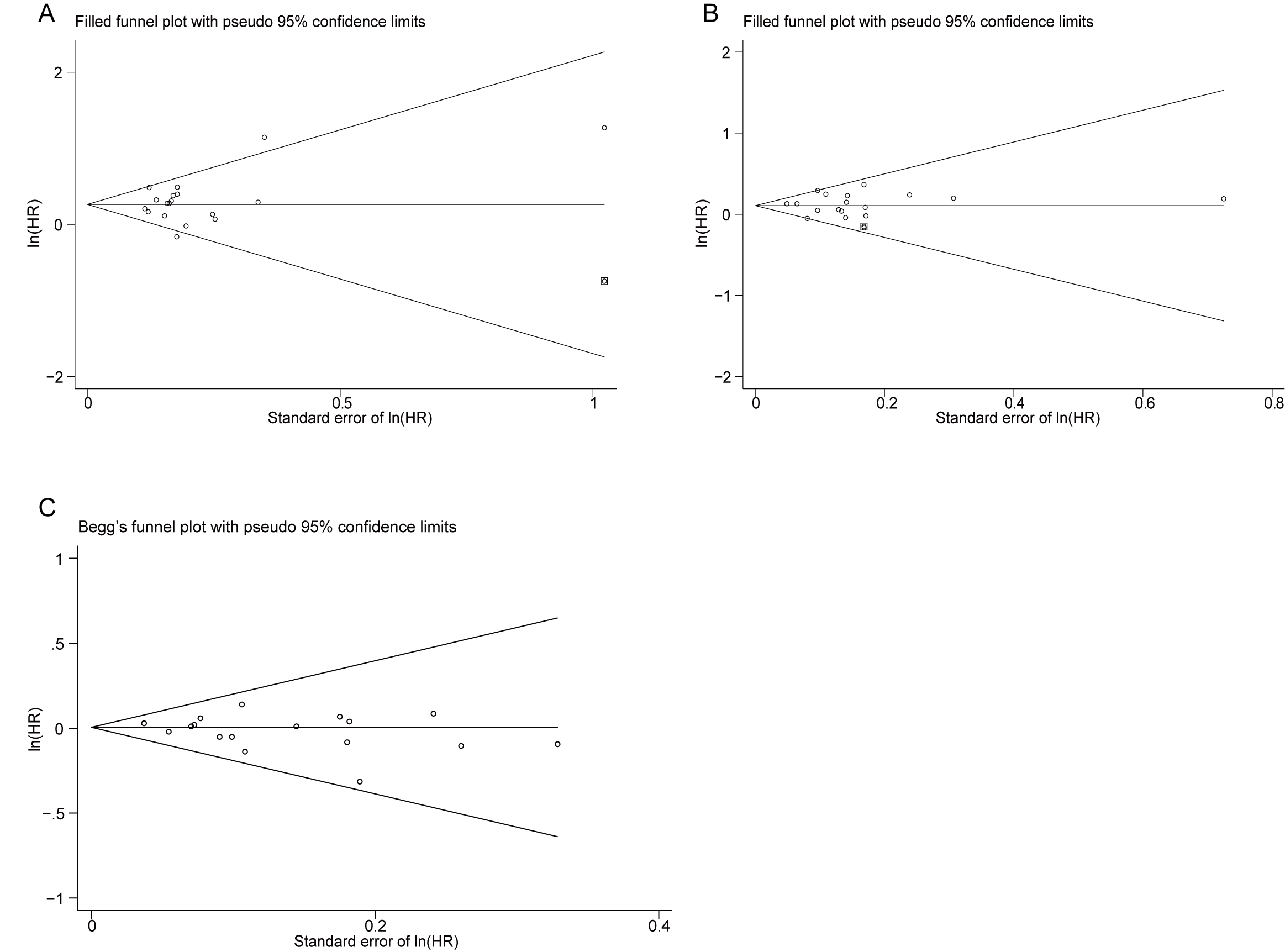

Supplement: Supplementary Figure 1 — Funnel plots for alcohol consumption and AF. (A) high alcohol intake, filled; (B) moderate alcohol intake, filled; (C) low alcohol intake. [file Image_1.TIF]

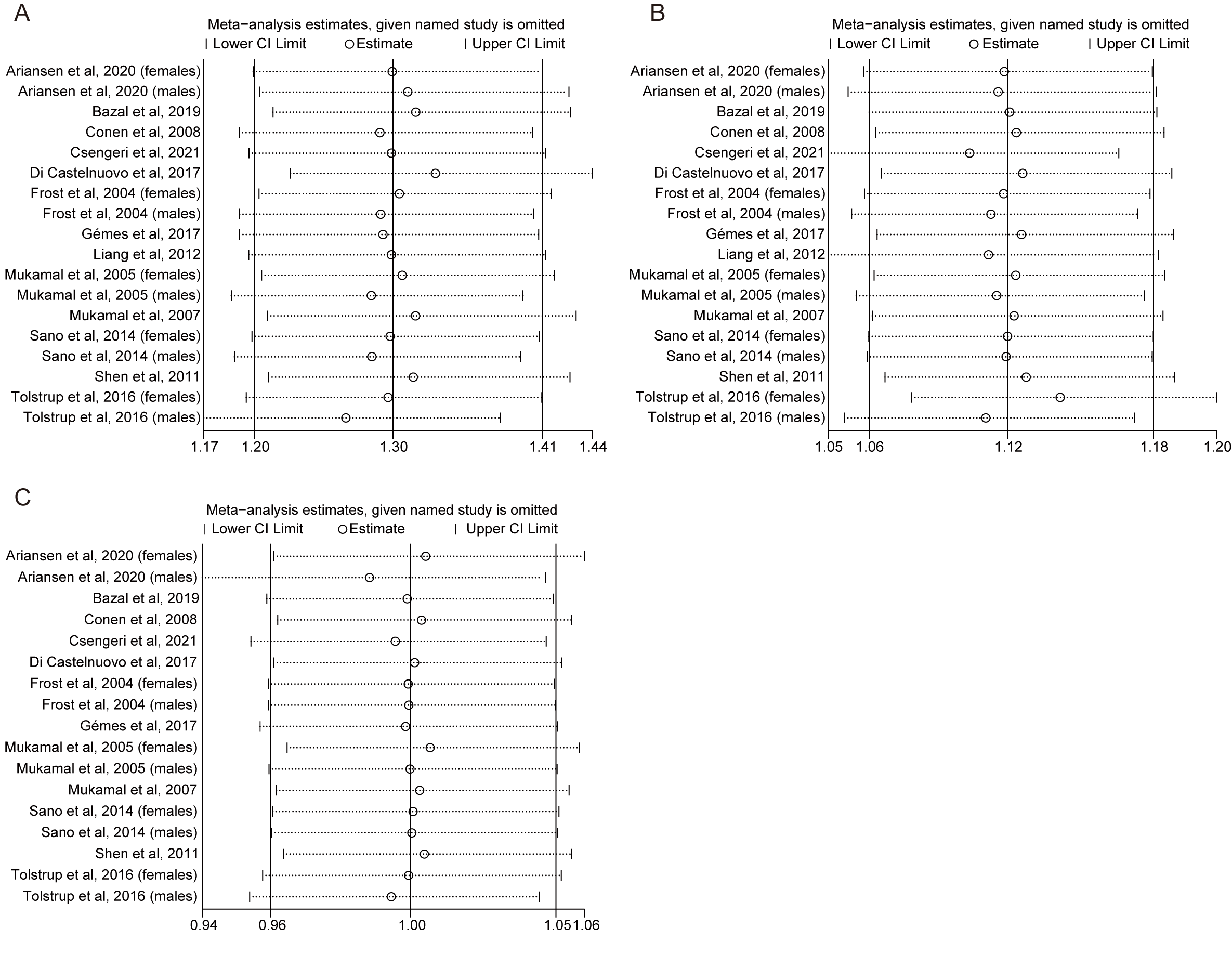

Supplement: Supplementary Figure 2 — Sensitivity analysis using the one-study removal method. (A) high alcohol intake; (B) moderate alcohol intake; (C) low alcohol intake. [file Image_2.TIF]
